# TSSP‐UNet: A Two‐Stage Weakly Supervised Pathological Image Segmentation With Point Annotations

**DOI:** 10.1049/syb2.70055

**Published:** 2026-03-03

**Authors:** Shaoqiang Wang, Guiling Shi, Yuchen Wang, Qiang Li, Yawu Zhao, Xiaochun Cheng

**Affiliations:** ^1^ Qingdao University of Technology Qingdao China; ^2^ Peking University People's Hospital Qingdao China; ^3^ Shandong University of Traditional Chinese Medicine Jinan China; ^4^ Computer Science Department Bay Campus Fabian Way Swansea University Swansea UK

**Keywords:** image segmentation, machine learning, neural network

## Abstract

Deep convolutional neural networks have demonstrated remarkable effectiveness in image segmentation. However, segmentation becomes challenging when training on images with complex instances. Moreover, obtaining annotations for high‐precision data is also difficult. Weakly supervised learning can address this issue by using nonspecialised annotations or supervised information from segmentation algorithms. In this study, we proposed TSSP‐UNet: a two‐stage weakly supervised segmentation approach. In the first stage, we trained a segmentation network augmented with constraint and attention mechanisms. These mechanisms are designed to operate on boundaries and superpixels generated from pseudo‐labels. For the attention network, two pseudo‐labels were used with a binary mask to add contour information to the segmentation process. Furthermore, a feature aggregation segmentation network was applied to the prominent foreground area in the image by incrementally adding elements. In the second stage, a refined confident learning algorithm improved the pseudo‐labels at the pixel level and then TSSP‐UNet was retrained using the modified superpixel labels. Testing on the MoNuSeg and TNBC datasets demonstrates that the approach performs well in the weakly supervised cell nucleus segmentation task compared with baseline methods.

## Graphical abstract

Deep convolutional neural networks excel at image segmentation but face challenges with complex instance training and high‐precision annotation acquisition. This study proposes TSSP‐UNet, a two‐stage weakly supervised segmentation approach: the first stage trains a segmentation network with constraint and attention mechanisms plus a feature aggregation network, whereas the second stage refines pseudo‐labels via a confident learning algorithm before retraining. Tests on the MoNuSeg and TNBC datasets show it outperforms baseline methods in weakly supervised cell nucleus segmentation.

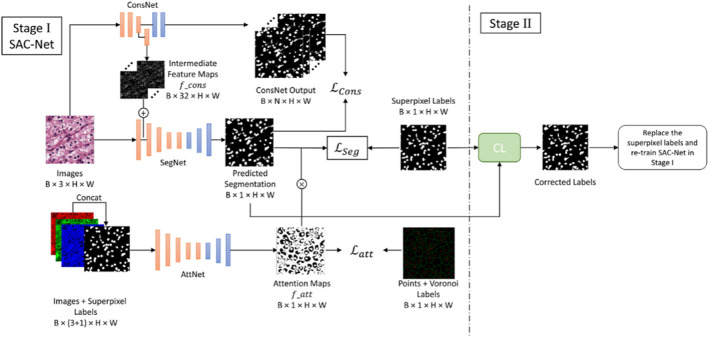

## 1 Introduction

Histopathological diagnosis is widely recognised as the ‘gold standard’ for cancer confirmation [1, 2, 3]. With the rapid advancement of digital pathology, computer‐aided diagnosis (CAD) systems have become increasingly important in clinical workflows [4, 5]. In these systems, cell nucleus segmentation is a fundamental and critical task as its results directly influence the accuracy of subsequent tumour grading, prognosis assessment and other quantitative analyses [6, 7, 8, 9]. Although fully supervised deep convolutional neural networks (CNNs) have achieved significant performance improvements in pathological image segmentation in recent years [10, 11, 12], these methods typically require massive amounts of fine‐grained pixel‐level annotations. However, due to the complex structures, diverse morphologies and frequent overlapping of cells in pathological images, obtaining high‐quality pixel‐level annotations is not only time‐consuming and labour‐intensive but also requires the involvement of professional pathologists, which severely limits the large‐scale application of fully supervised models in real‐world medical scenarios.

To alleviate the dependency on pixel‐level annotations, weakly supervised learning (WSL) has become a current research hotspot [4, 5, 13]. Unlike fully supervised methods, WSL aims to utilise sparse annotations that are easier to acquire—such as image‐level labels, bounding boxes, scribbles or point annotations—to train segmentation models. Among these annotation forms, point annotation is widely adopted in cell nucleus segmentation tasks due to its ability to provide specific location information of target objects at an extremely low cost. Existing studies typically utilise point annotations combined with Voronoi diagrams or superpixel algorithms to generate pseudo‐labels and subsequently train segmentation networks in a fully supervised manner. For instance, Qu et al. proposed a weakly supervised method based on point annotations and Voronoi diagrams, achieving rapid localisation and segmentation of cell nuclei [14, 15]; other researchers have also explored using scribbles or multitask learning to optimise segmentation results [16, 17, 18].

Despite the progress made by existing weakly supervised methods, directly training with generated pseudo‐labels remains significantly flawed [19, 20]. The core issue lies in the fact that pseudo‐labels generated from sparse point annotations often contain substantial noise. For example, methods based on Voronoi diagrams typically assume that nucleus boundaries are entirely located within their Voronoi cells, ignoring background regions; meanwhile, in tumour regions with dense cells, simple geometric partitioning often fails to accurately fit real cell boundaries [21, 22, 23]. Such noise and inaccurate boundary information in pseudo‐labels can mislead network training, causing the model to produce blurred boundaries, adhesion or misclassification during prediction. Therefore, effectively suppressing pseudo‐label noise and extracting robust features in the absence of precise pixel‐level ground truth is the primary challenge currently facing weakly supervised cell nucleus segmentation.

The first challenge this paper addresses is how to design a network mechanism that can still focus on real cellular structural features under the supervision of noisy pseudo‐labels. Relying solely on a single segmentation network makes it difficult to distinguish between noise and real signals. Inspired by the human visual attention mechanism, we believe that introducing additional structural priors can help the network ‘focus’. Although superpixels are generated unsupervised, they can fit local image boundaries well based on colour and texture similarity [24]. If we can combine the geometric structure information provided by superpixels with the feature extraction capability of deep networks, utilising an attention mechanism to suppress uncertain boundary regions in pseudo‐labels although using a constraint mechanism to force the network to preserve low‐level texture information, the model's robustness to noise will be significantly enhanced.

The second challenge this paper addresses is how to fundamentally improve the quality of supervision signals, that is, achieving active correction of pseudo‐labels. Most existing methods passively adapt to noisy labels, ignoring that the labels themselves can be optimised. In fact, deep learning models generate prediction probabilities during training, and this probability distribution often contains confidence information about the samples. We believe that the model's own prediction confidence can be used to identify errors in pseudo‐labels. By introducing a confident learning (CL) strategy [25], we can screen out high‐probability error markers based on the joint distribution of prediction probabilities and given labels and correct them. This ‘generate‐correct‐retrain’ closed‐loop strategy can progressively improve the accuracy of pseudo‐labels, thereby further enhancing segmentation performance.

Based on the aforementioned motivations, this paper proposes a two‐stage weakly supervised pathological image segmentation framework based on point annotations, termed TSSP‐UNet (Two‐stage Weakly Supervised Pathological Image Segmentation). The framework consists of two core stages: In the first stage, we construct a joint architecture (SAC‐Net) comprising a segmentation network (SegNet), an attention network (AttNet) and a constraint network (ConsNet). AttNet utilises superpixels and Voronoi labels to generate attention maps that guide SegNet to focus on high‐confidence foreground regions; ConsNet extracts low‐level features in parallel to constrain the structural consistency of the segmentation results. In the second stage, we introduce the confident learning (CL) algorithm [25] to clean and correct the noise in the pseudo‐labels generated in the first stage. The corrected labels are then used to retrain SAC‐Net, achieving finer pixel‐level segmentation.

The main contributions of this paper are summarised as follows:

1. We propose a novel two‐stage weakly supervised segmentation framework (TSSP‐UNet) that achieves high‐quality cell nucleus segmentation in pathological images using only sparse point annotations, effectively resolving the annotation difficulty.
2. We designed the SAC‐Net architecture, which effectively utilises the structural information of superpixels and the low‐level texture features of images by integrating an attention network (AttNet) and a constraint network (ConsNet), significantly enhancing the model's feature extraction capability under noisy labels.
3. We innovatively introduce confident learning into the weakly supervised segmentation task, proposing a label denoising and correction strategy that further improves the model's segmentation accuracy by iteratively optimising pseudo‐label quality.
4. Experimental results on two public datasets, MoNuSeg and TNBC [26, 27], demonstrate that this method performs excellently under weakly supervised settings, with segmentation performance being competitive and even approaching that of fully supervised methods.

## 2 Methodology

### 2.1 Pseudo Label Generation Method

Since fully supervised segmentation models rely on expensive pixel‐level annotations, this study aims to explore training high‐precision segmentation networks using only low‐cost sparse point annotations. However, existing deep CNNs typically require dense pixel‐wise masks to calculate loss functions and update weights. To bridge the gap between sparse point annotations and the requirement for dense predictions, we transform point annotations into two types of pseudo‐labels with complementary characteristics—Voronoi labels and superpixel labels—to construct preliminary supervision signals.

First, to establish the ‘existence’ of nuclei and address cell adhesion in dense regions, we construct Voronoi labels using the positional information of point annotations. By applying a Euclidean distance transform to the centroids of cell nuclei, the image space is partitioned into multiple polygonal regions, ensuring that each region contains exactly one nucleus centroid [28]. This partition provides a strong global topological prior, enforcing separation between instances. However, Voronoi labels have significant limitations: their linear boundaries generated based on geometric distance often fail to fit the varying organic shapes of real nuclei and inevitably misclassify surrounding background as foreground, thereby introducing significant background noise [15].

To compensate for the deficiencies of Voronoi labels in boundary delineation, we further introduce superpixel labels based on the simple linear iterative clustering (SLIC) algorithm [24]. Leveraging local colour similarity and texture consistency, the SLIC algorithm clusters pixels into superpixel blocks that closely adhere to edges. Strategically, we label superpixel blocks containing point annotations as foreground (nuclei), whereas the rest are treated as background. Unlike Voronoi labels, superpixel labels keenly capture local structural features, significantly improving the boundary adherence of pseudo‐labels.

The two aforementioned types of pseudo‐labels form a significant complementary effect functionally: Voronoi labels ensure global topological separation, whereas superpixel labels provide local edge details. Nevertheless, utilising them directly as ground truth for training still poses severe challenges as Voronoi labels contain background noise, and unsupervised superpixels may suffer from oversegmentation or undersegmentation errors in low‐contrast regions. This inherent noise in supervision signals motivated our design of the subsequent SAC‐Net architecture (incorporating attention and constraint mechanisms) and the confident learning strategy [25] in the second stage, aiming to dynamically suppress noise and iteratively refine labels during training to achieve robust segmentation performance.

### 2.2 Overview of TSSP‐UNet

**FIGURE 1:**
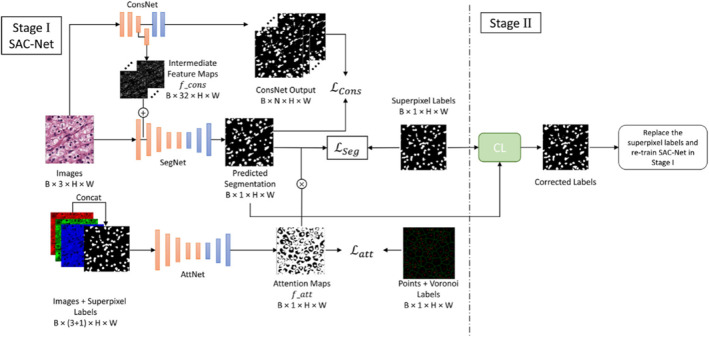
General framework of SAC‐Net.

As illustrated in Figure 1, to achieve high‐precision cell nucleus segmentation in the absence of pixel‐level ground truth, we propose the TSSP‐UNet framework. This framework is designed not as a simple stacking of modules but as a cascaded system consisting of two stages with complementary functions. It aims to progressively address the noise issue in weakly supervised signals through a strategy of ‘noise‐robust feature extraction first, followed by active label refinement’.

In the first stage, our core objective is to train a robust model capable of capturing key structural features of cell nuclei under the supervision of noisy pseudo‐labels. To this end, we constructed the SAC‐Net (segmentation, attention and constraint network). SAC‐Net accepts raw images along with Voronoi and superpixel pseudo‐labels generated from point annotations as input. Considering the imperfection of pseudo‐labels, we do not force the segmentation network (SegNet) to directly fit all labels. Instead, we adopt a soft constraint strategy: utilising weight maps generated by the attention network (AttNet) to guide the model to focus only on high‐confidence foreground regions; simultaneously, a parallel constraint network (ConsNet) extracts low‐level image features as regularisation constraints, preventing the model from losing original texture and boundary information whilst fitting noisy labels.

Although the model in the first stage possesses strong noise robustness, its performance ceiling is ultimately limited by the quality of the pseudo‐labels themselves. To break this bottleneck, the second stage focuses on actively refining the supervision signals. We introduce the confident learning (CL) algorithm, utilising the SAC‐Net trained in the first stage to predict the training data. By analysing the joint distribution between the model prediction probability and the initial noisy labels, we can identify pixel samples that the model considers mislabelled with high confidence. Subsequently, we flip and correct these noisy labels to generate higher‐quality refined labels and use this cleaned data to retrain SAC‐Net. This two‐stage closed‐loop design allows TSSP‐UNet to automatically optimise label quality and significantly improve final segmentation accuracy without manual intervention.

### 2.3 SegNet Segmentation Network and AttNet Attention Network

**FIGURE 2:**
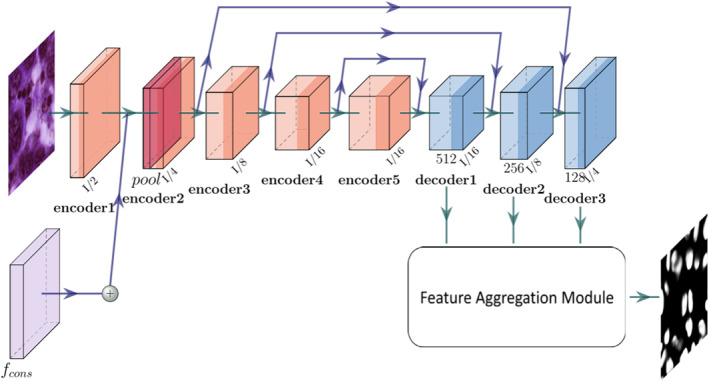
The SegNet framework.

To better understand the complex features of the cell nucleus, the feature aggregation SegNet segmentation network has been designed. It comprises encoder and decoder structures, along with additional feature aggregation modules, as illustrated in Figure 2. The original image serves as the input, and the generated superpixel pseudo‐label (*y*_*s*_) is used as supervision for training the segmentation network. The network is tailored to accommodate the complexity of the cell nucleus, aiming to achieve initial segmentation results.

In Figure 9, He et al. [29] utilise ResNet‐50 in the encoder. The last four blocks are connected to the decoder to reduce computation resulting from the spatial dimension. Xu et al. [30] segmented WSI images using a combined architecture that integrates both U‐Net and ResNet structures. Furthermore, the intermediate feature map from ConsNet, referred to as fcons, is merged with the feature map produced by encoder1. Element‐wise addition is used to combine these maps, and the combined results are then passed to subsequent layers. Deformable convolution is implemented in each encoding layer to address the limitations of fixed geometric transformations inherent in the convolutional building block [31]. In deformable convolution, an offset is applied to each sample point during the convolution operation. Additional convolutional layers are employed to enable the network to more effectively utilise spatial information for feature representation. Additionally, the dilation strategy is applied in the last encoder block, with a dilation rate of 2 and an output stride of 1/16. This approach facilitates an increase in the receptive field size whilst preserving image resolution.

**FIGURE 3:**
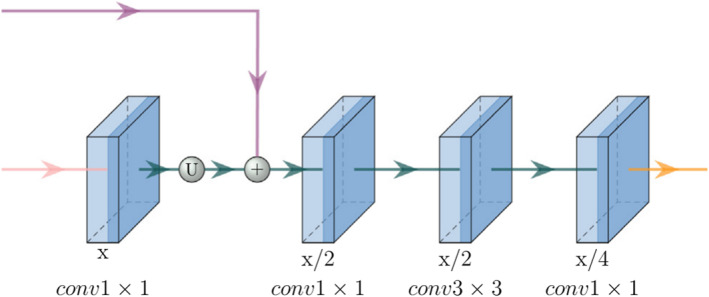
The decoder structure.

A traditional decoder layer connects feature maps from two levels, followed by a deconvolution layer to recover feature resolution. However, it may retain redundant features from previous layers, thereby increasing computational complexity. Therefore, a more efficient decoder block has been designed as illustrated in Figure 3.

**FIGURE 4:**
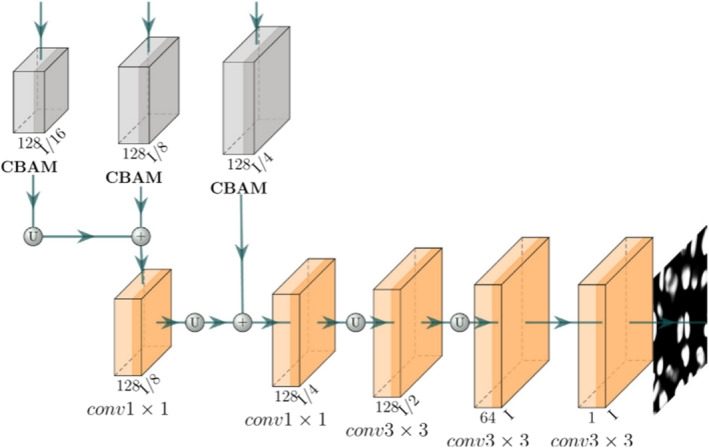
Structure of the feature aggregation module.

A 1 × 1 convolutional layer is first applied to the output of the previous layer as a transformation. Subsequently, feature maps of the same shape are sampled from the lower encoder and merged using element‐by‐element addition. Weighted merging of feature maps is employed to sample through 1 × 1, 3 × 3 and again 1 × 1 convolutional layers. Moreover, the feature maps from each decoder block have different scales and not all levels of features contribute equally to the final output. Consequently, an aggregation module is attached to the decoder block to produce a segmented output. This aggregation module combines channel and spatial attention layers, incorporating the convolutional block attention module [32]. It serves to highlight important information from the output of each decoder block for improved aggregation as depicted in Figure 4.

The feature maps from the three decoder blocks are passed through the three corresponding CBAM blocks. The outputs of the CBAM are merged by element‐by‐element addition and fed through an upsampling operation to obtain the final predicted probability maps. CBMA is an attention mechanism commonly used in computer vision modelling. It can help enhance intermediate features by using the product of a one‐dimensional channel attention map and a two‐dimensional spatial attention map to improve the performance of the model. The CBAM model uses two submodules, channel attention and spatial attention. The channel submodule obtains two pooling results by performing maximum pooling and average pooling on the input feature maps, respectively, and inputs them to a shared network to generate a channel attention map. This shared network consists of a multilayer perceptron (MLP) and a hidden layer. Their outputs are combined by element‐by‐element addition to obtain a comprehensive channel attention graph. The spatial submodule performs a similar operation on the feature maps along the channel axis and feeds them into the convolutional layer to produce the corresponding spatial attention maps. The CBAM applies the channel and spatial attention submodules in turn to assist the flow of information within the network by learning to emphasise or suppress information. In order to train SegNet, the binary cross entropy loss is chosen as *L*_seg_ to optimise SegNet according to Equation (1).

(1) $$L_{\text{seg}}=L\left(f\left(I\right)⊙f_{\text{att}},y_{s}\right),$$

where *f*, *I* and ⊙ denote SegNet, input image and element‐by‐element multiplication, respectively. Each vector predicted by SegNet is multiplied element‐by‐element with the corresponding vector *f*_att_ from the output of the attention network. The purpose of the attention network is to guide the segmentation of SegNet during the training process to achieve more accurate segmentation results.

Super pixel labels can capture rich contour information in an image. However, a high degree of labelling noise exists in such labels. In the work, an attention network (AttNet) is designed instead of clean labels from pseudo‐labels, which is considered as a data‐dependent denoising method. The attention feature maps generated by the attention network help identify the true labels in *y*_*s*_, directing the Segmentation network to focus on these regions. AttNet generates a binary mask that separates the central kernel region and the background from the noisy labelled regions around the kernel boundaries. Multiplying this binary mask with the segmentation output of SegNet element‐by‐element focuses the training of SegNet on more confident regions.

As shown in Figure 1, the input images and their corresponding super pixel labels are concatenated to form 4 channels input to AttNet. The super pixel channels provide additional structural information in the image, in particular highlighting contours. The training of AttNet is supervised by point and Voronoi labels that indicate real nuclei and background regions. As a result, the output of AttNet confidently identifies regions classified as nuclei or background, masking regions around nuclei boundaries as noisy regions. AttNet efficiently assigns pixel weights to the output of SegNet to improve segmentation learning. The attention network uses the same architecture as the Segmentation network, with the smaller ResNet‐18 as an encoder. ResNet‐18 is only used to extract features for simplifying the aggregation module and does not require more computational time during training. To train AttNet, the partial element‐by‐element loss between AttNet output (*f*_att_) and Voronoi ground‐truth label (*y*_*v*_) is calculated by Equations (2) and (3).

(2) $$f_{\text{att}}=g\left(c\left(I,y_{s}\right)\right)$$

(3) $$L_{\text{att}}\left(f_{\text{att}},y_{v}\right)=\frac{\sum_{y\in y_{v}^{+}}{|1-f_{\text{att}}|}_{1}}{\left|y_{v}^{+}\right|}+\alpha \cdot \frac{\sum_{y\in y_{v}^{-}}{\left|1-f_{\text{att}}\right|}_{1}}{\left|y_{v}^{-}\right|}$$

where *g*, *y*_*v*_^+^, *y*_*v*_^−^, |*y*_*v*_^+^| and |*y*_*v*_^−^| represent the total number of positive and negative labels for the output of the attention network, positive labels, negative labels and Voronoi labels of the image *I*, respectively. *c* (*I*, *y*_*s*_) is the cascade operation. *α* is a constant that balances positive and negative labels.

### 2.4 ConsNet Constraint Network

**FIGURE 5:**
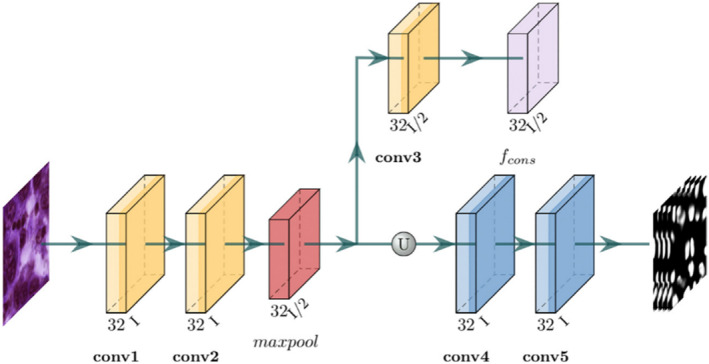
Framework of ConsNet.

To further improve the segmentation performance, a shallow constraint network (ConsNet) is designed which can run in parallel with SegNet. ConsNet is a shallow CNN that extracts low‐level information [33]. The feature maps from ConsNet are also effectively combined into SegNet as attention maps to highlight foreground regions. As shown in Figure 5, the original image is input into ConsNet. To emphasise the details of the input image, two convolutional layers (conv1 and conv2) are used to learn the local details. In the main branch, the feature maps are obtained through an upsampling operation and two more convolutional layers (conv4 and conv5) to find the final output of ConsNet.

The intermediate data are processed through another convolutional layer (conv3) to generate the intermediate feature map (*f*_cons_). *f*_cons_ is merged into SegNet by an element‐by‐element addition operation. The output *f*(*I*) of SegNet is used as supervision. To avoid generating redundant feature maps, conv1‐4 in ConsNet consist of the 3 × 3 kernel with 32 channels, followed by a BatchNorm layer and ReLU activation. conv5 has only a 3 × 3 kernel with the N channel. These shallow network layers can maintain the low‐level features of the original image, combined with the high‐level features for predicting the segmentation results. For the loss function, *L*_cons_ is used to train ConsNet and the output of SegNet, *f*(*I*), is used as the truth value. *L*_cons_ is shown in Equation (4).

(4) $$L=\frac{\sum_{n=1}^{N}\left|f\left(I\right)-{h\left(I\right)}_{n}\right|}{N}$$

where *h*(*I*)_*n*_ is the *n*th channel data output by ConsNet and |⋯| is the *L*_1_ loss. We employ the L1 loss for ConsNet instead of the standard MSE (L2) loss. Since pseudo‐labels inherently contain boundary noise, MSE would heavily penalise outliers, causing overfitting to incorrect annotations. In contrast, L1 loss produces sparser gradients and is more robust to these outliers. Furthermore, unlike the SegNet branch which focuses on semantic abstraction, ConsNet is explicitly designed as a shallow parallel branch to preserve low‐level high‐frequency details (texture and boundaries), which are often lost in deep encoders. In order to train SegNet, AttNet and ConsNet simultaneously, the total loss *L*_total_ is defined as a linear combination of *L*_seg_, *L*_att_ and *L*_cons_ as shown in Equation (5).

(5) $$L_{\text{total}}=\beta {\cdot L}_{\text{seg}}+\gamma \cdot L_{\text{att}}+\delta \cdot L_{\text{cons}}$$

where *β*, *γ* and *δ* are utilised as hyperparameters guiding *L*_seg_, *L*_att_ and *L*_cons_, respectively. These values were determined through a grid search on the validation set to effectively balance the trade‐off between segmentation accuracy and boundary consistency.

### 2.5 Confident Learning Algorithm for Noise Label Correction

After SAC‐Net segmentation, the segmentation results are still inaccurate due to the supervision of pseudo labels. Therefore, the confident learning (CL) algorithm is further applied to refine the super pixel label (*y*_*s*_) [25]. Then, the whole SAC‐Net is trained again using the improved super pixel label (*y*_*re*_). This process is defined as the second stage in our model. It is assumed that the image data *X* = {(*x*_0_, *y*_*s*0_),…, (*x*_*n*_, *y*_*sn*_)} is labelled as class a. A label is considered to contain noise if the data are predicted to belong to another class *b*. The prediction probability is higher than the threshold of class a. The label is considered to contain noise. To identify labelling errors, CL estimates the joint distribution ($Q_{y_{s},y^{*}}$) between super pixel label (*y*_*s*_) and ground‐truth label (y*) First, the counting matrix ($C_{y_{s},y^{*}}$) is constructed as shown in Equations (6) and (7).

(6) $$C_{y_{s}=i,y^{*}=j}=\left|Z_{y_{s}=i,y^{*}=j}\right|$$

(7) $$Z_{y_{s}=i,y^{*}=j}=\left\{z\in X:y_{sz}=i,\hat{p}\left(y=j;z\right)>t_{j},j=\underset{k}{\mathrm{argmax}}\hat{p}\left(y=j;z\right)\right\}$$

where *j* is the class with the highest prediction probability among all classes for that pixel. *t*_*j*_ belongs to class *j* with the average prediction probability of all pixels. By normalisation, $Q_{y_{s},y^{*}}$ is calculated by Equation (8).

(8) $$Q_{y_{s},y^{*}}=\frac{\frac{C_{y_{s}=i,y^{*}=j}}{\sum_{j\in m}C_{y_{s}=i,y^{*}=j}}\cdot \left|X_{y_{s}=i}\right|}{\sum_{i\in m,j\in m}\frac{C_{y_{s}=i,y^{*}=j}}{\sum_{j\in m}C_{y_{s}=i,y^{*}=j}}\cdot \left|X_{y_{s}=i}\right|}$$

When the *y*_*s*_ and y* are obtained, the error labels are corrected. In the nondiagonal position of the count matrix, *n*·$Q_{y_{s},y^{*}}$ samples are extracted for correction and sorted by the maximum interval of $\hat{p}\left(y=j;x\right)-\hat{p}\left(y=i;x\right)$.

## 3 Results

### 3.1 Analysis of Super Pixel Pseudo Labels and Voronoi Unit Images

**FIGURE 6:**
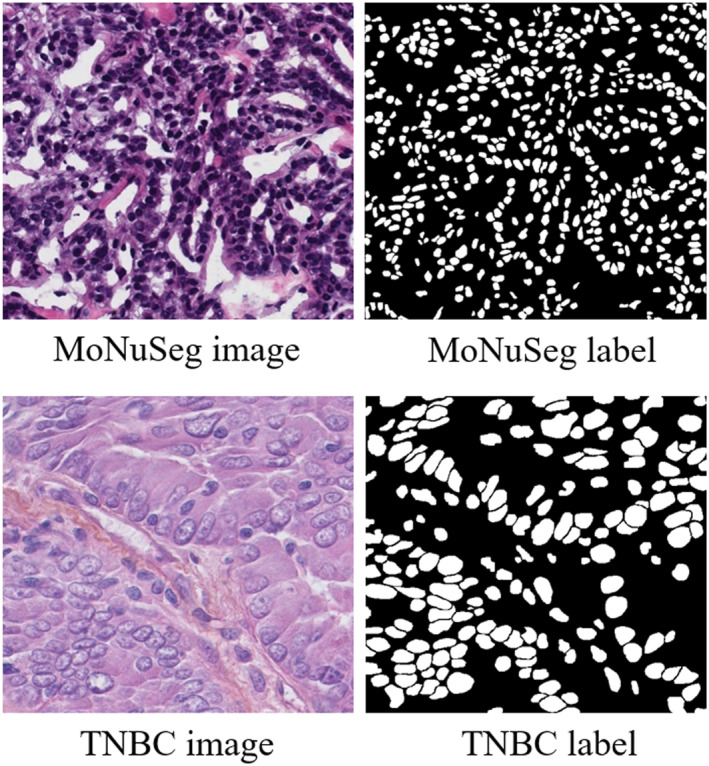
Display of sample data.

The method proposed in this paper is evaluated on two public datasets of histopathological images used for cell nucleus segmentation, including MoNuSeg and TNBC [26, 27]. The TNBC dataset is a publicly available dataset for breast cancer classification studies. The images in the dataset are derived from breast tissue specimens from different patients. Each image can be classified into three types: tumour, normal tissue and lymph node. These datasets are provided with pixel‐by‐pixel ground‐truth annotations. MoNuSeg contains 51 images from a total of seven different organs. Each image is 1000 × 1000 pixels. TNBC contains 50 images from 11 different patients. Each image is 512 × 512 pixels. These models are implemented using the Pytorch framework and trained on 12 Intel Xeon Silver 4114 CPUs and 4 GeForce‐RTX‐2080‐Ti GPUs. The Adam optimiser is used in the code with the learning rate set to 0.001 and the weight decay set to 0.0005. The learning rate is halved when the *L*_total_ of the validation set does not decrease within 4 epochs. The training process consists of 60 epochs, with 1 batch set trained in each period in MoNuSeg and 2 batch sets trained in each period in TNBC. Data enhancement includes Gaussian blurring, hue and saturation adjustment, affine transformation, horizontal and vertical flipping [34]. Bilinear upsampling is used for the upsampling operation. The hyperparameters *α*, *β*, *γ* and *δ* are set to 0.4, 0.8, 1 and 0.4, respectively. In addition, a number of ablation experiments are performed. Sample raw pathology images and corresponding labelled files from the MoNuSeg and TNBC datasets are shown in Figure 6.

**FIGURE 7:**
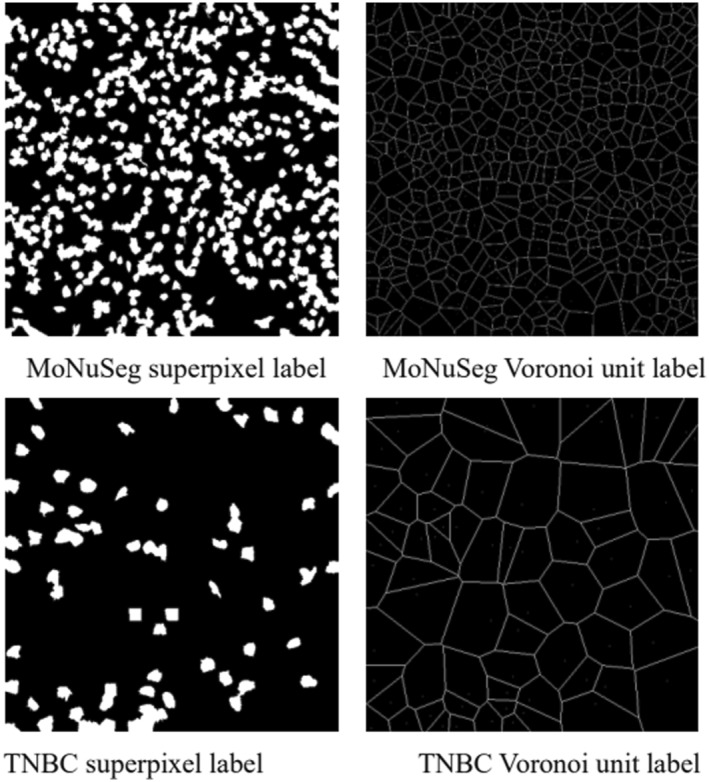
Super pixel pseudo labels and Voronoi unit images.

The super pixel and Voronoi unit image labels are obtained by using SLIC algorithm and Euclidean distance transform as shown in Figure 7.

**FIGURE 8:**
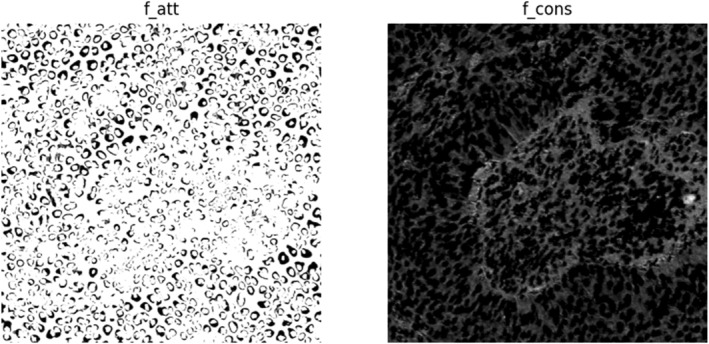
Attention network and constraint network output feature images.

The generated super pixel pseudo labels are flawed. With the help of the above two pseudo labels, the two types of images are fed into the network and fusion features are extracted. The output feature maps and segmentation result maps of each module are obtained as shown in Figure 8.

**FIGURE 9:**
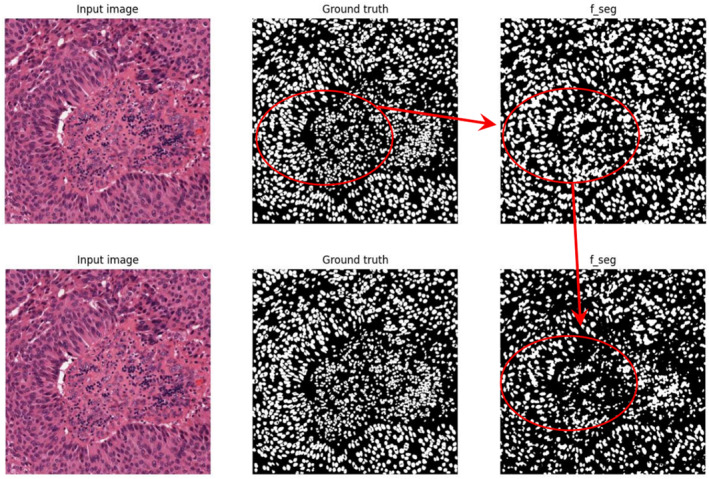
Segmentation comparison results after CL correction for super pixel pseudo labels.

### 3.2 Treatment Through Confidence Learning

The initially generated super pixel pseudo labels carry a lot of noise. After processing by the attention network and constraint network, the segmentation attention is more focused on the region of interest. The low‐level features extracted by the constraint network reduce the noise of the super pixel pseudo labels. However, not all pseudo labels in the generated pseudo labels are reliable. Some of the pseudo‐labels have subversive errors with a certain probability. So, confidence learning (CL) is used for correcting super pixel pseudo labels. The re‐training results are shown in Figure 9.

**FIGURE 10:**
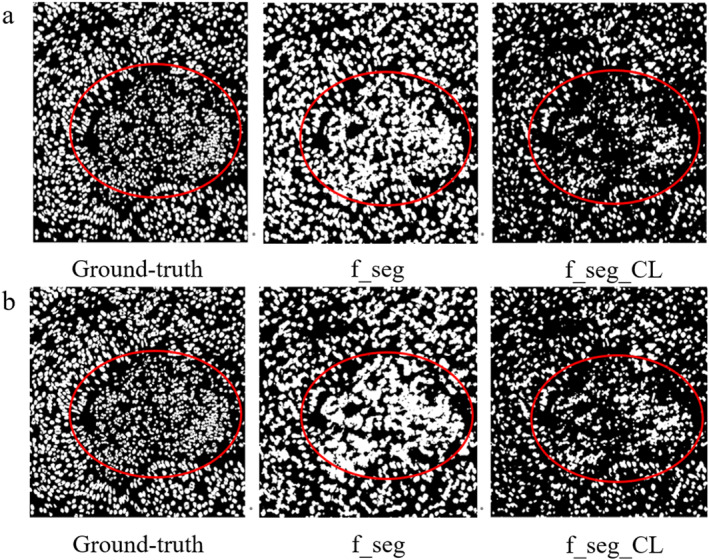
CL‐corrected pre and postsegmentation images after the centre of mass randomly shifted by: (a) 3 pixels and (b) 5 pixels.

The improvement of segmentation results after CL processing of generated super pixel pseudo labels is tremendous. The improvement of segmentation results after one CL processing is the most significant. When the number of CL‐processed super pixel pseudo labels is greater than or equal to 2, the output feature map results of the attention network deteriorate and contain fewer features from the original image. This makes it more difficult for the segmentation network to focus its attention on the region of interest. During the generation of the Voronoi cell map, *r* = 0 denotes the point annotation of centre of mass. *r* is randomly shifted by 3–5 pixel points which indicates noisy point annotations with random shifts and the results are shown in Figure 10.

**FIGURE 11:**
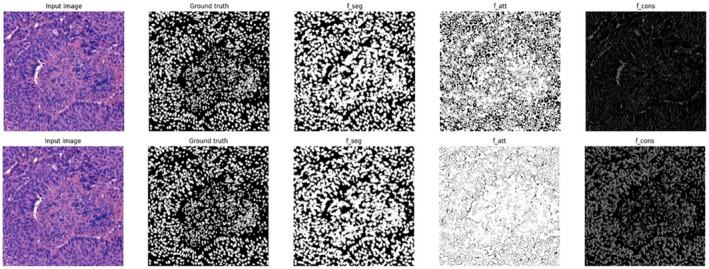
Segmentation results after data colour normalisation.

The image is processed using the colour normalisation method and pseudo labels are generated for the experiment as presented in Figure 11. After CL processing, the segmentation results are not significantly improved. Attention network and constraint network output feature maps changed. The feature maps of the attention network that did not undergo CL processing were able to focus more on the region of interest for image segmentation. The constraint network with CL processing contains more shallow features of the image. Both affect the image Segmentation results.

### 3.3 Change in Total Loss

**FIGURE 12:**
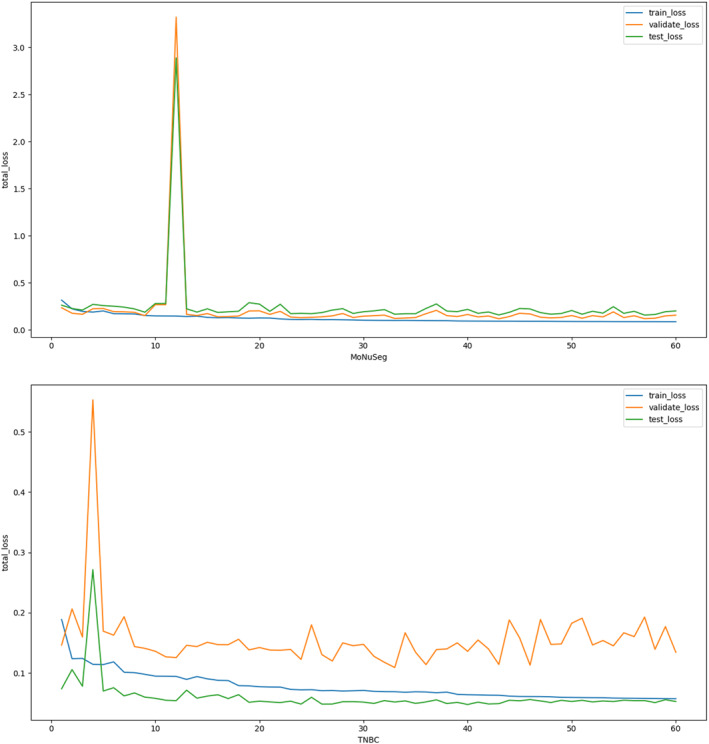
Change of total loss during training of the two datasets.

Figure 12 shows the trend of total loss during iterative segmentation generation for both dataset labels after using CL. The changes in loss for the training and testing process are in line with expectations, but the changes in validation loss for TNBC fluctuate a lot. It is worth noting that the transient loss spike in the TNBC dataset was attributed to a specific mini‐batch containing hard examples with conflicting pseudo‐label information. However, as shown in the graph, the loss curve rapidly decreased and reconverged immediately after the spike. This demonstrates the resilience of our model: despite momentary gradient shocks, the regularisation from ConsNet prevented divergence and allowed the model to stabilise.

The IoU and Dice for several experiments are displayed in Table 1. The results of the experiments that underwent CL treatment were generally better than those that did not. The experimental results for the dataset norm are better than those of the first three methods, with an average improvement of at least 1% in IoU and Dice.

**TABLE 1:** Summary of experimental results.

Annotations	MoNuSeg				TNBC			
	IoU		Dice		IoU		Dice	
	Without CL	CL	Without CL	CL	Without CL	CL	Without CL	CL
Centroid (*r* = 0)	0.6474	0.6754	0.7860	0.8062	0.6185	0.6739	0.7642	0.8051
Shift (*r* = 3)	0.6609	0.6740	0.7959	0.8052	0.7197	0.6731	0.8370	0.8046
Shift (*r* = 5)	0.5905	0.6017	0.7425	0.7513	0.5819	0.5988	0.7357	0.7491
Dataset norm	0.6763	0.6874	0.8069	0.8148	0.7170	0.7064	0.8352	0.8279

### 3.4 Fully Supervised Cell Nucleus Segmentation Experiment Based on U‐Net Structure

**FIGURE 13:**
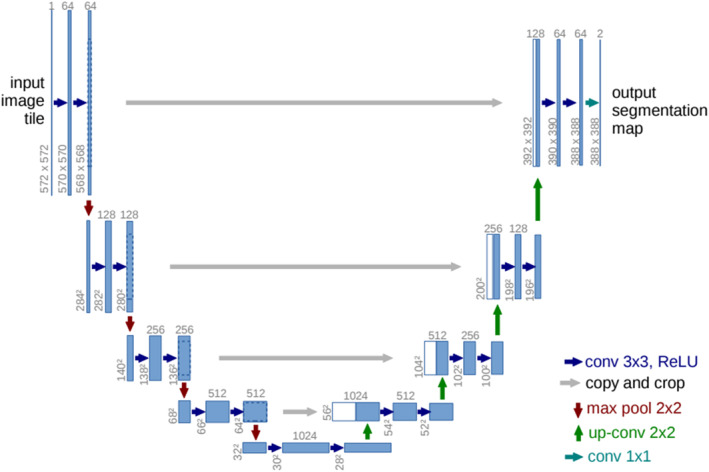
U‐Net structure.

Fully supervised cell nucleus segmentation experiments are conducted using the U‐Net structure through the self‐contained ground‐truth labels in both datasets as illustrated in Figure 13.

**FIGURE 14:**
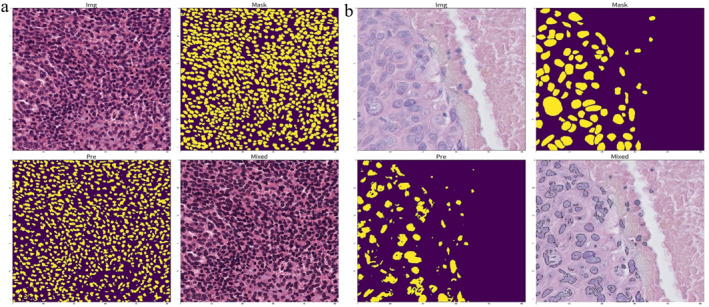
Segmentation results of the U‐Net framework on (a) MoNuSeg and (b) TNBC.

The segmentation accuracies obtained on the two datasets are 0.8545 and 0.9239, respectively. The segmentation accuracies on the TNBC dataset are higher than the accuracies on MoNuSeg. This may be due to the fact that the image size in MoNuSeg is 1000 × 1000. The excessive number of cells in it makes the segmentation of foreground and back view of the image more difficult. So, the segmentation effect is reduced. The U‐Net segmentation is shown in Figure 14.

The results of U‐Net segmentation are satisfactory. The results of the simplest fully supervised cell segmentation based on the U‐Net framework are better than the cell segmentation based on weakly supervised pseudo‐labelling. Weakly supervised pseudo‐labelling‐based cell segmentation still requires in‐depth research, which can reduce the time and effort spent on data labelling.

## 4 Conclusions

In this work, a weakly supervised approach was proposed to segmentation histopathological images of cell nuclei by introducing TSSP‐UNet. First, a segmentation network combining the attention mechanism and low‐level feature constraints is trained based on point labels, Voronoi labels and super pixel labels. The second stage applies a confidence learning algorithm to refine the segmentation output. Pseudo points labels, Voronoi labels and super pixel labels are considered as noisy labels and various methods are used to solve this problem. Attention networks in the first stage focus training on more confident regions. Constraint networks are used to highlight low‐level structural information. Confidence learning in the second stage is used to modify the labels explicitly. Experimental results on two histopathology image datasets show that the segmentation results are close to the fully supervised U‐Net cell nucleus segmentation. The method of this work has achieved better results. Moreover, a limitation of our approach is that the Voronoi assumption may lead to boundary truncation in dense tumour regions (e.g., in TNBC) where nuclei are tightly clustered. Although our superpixel refinement alleviates this, future work will incorporate instance‐level separation logic to better handle these crowded scenarios Therefore, the existing weakly supervised segmentation methods in histopathology images need to be further improved. This facilitates the researchers to acquire the centre of mass points to predict the segmentation results without labelling the images. In addition, the acquisition of cellular points is still a difficult challenge that values more in‐depth exploration.

## Author Contributions

**Shaoqiang Wang:** writing – original draft, validation, software, methodology, data curation. **Guiling Shi:** software, formal analysis. **Yuchen Wang:** validation, supervision. **Qiang Li:** methodology, formal analysis. **Yawu Zhao:** formal analysis, software, methodology, data curation. **Xiaochun Cheng:** formal analysis, software, methodology, data curation.

## Funding

This work was funded by UKRI Grant EP/W020408/1 and Grant RS718 through Doctoral Training Centre at Swansea University.

## Conflicts of Interest

The authors declare no conflicts of interest.

## Data Availability

The data that support the findings of this study are available from the corresponding author upon reasonable request.
